# Safety Evaluation of Large-Scale Administration of a Novel Human Diploid (SV-1) Cell Line-Derived Varicella Attenuated Live Vaccine in Children 7–12 Years Old

**DOI:** 10.3390/vaccines14010019

**Published:** 2025-12-23

**Authors:** Yuanyuan Zhu, Yurong Li, Jing Yu, Borong Xu, Xun Li, Ran Hu, Xiaozhe Song, Yonghong Sun, Dongsheng Liu, Yuan Ren, Xiang Sun, Zhiguo Wang

**Affiliations:** 1 Department of Immunization Program, Jiangsu Provincial Center for Disease Control and Prevention, Nanjing 210009, China; zyy2018@foxmail.com (Y.Z.); yujingyuyan@163.com (J.Y.); jscdcxbr@163.com (B.X.); 18020183211@163.com (X.L.); huhu1989acer@126.com (R.H.); 2 Sinovac Holding Group Co., Ltd., Beijing 100085, China; liyr8134@sinovac.com (Y.L.); renyuan@sinovac.com (Y.R.); 3 Department of Immunization Program, Xuzhou Center for Disease Control and Prevention, Xuzhou 221006, China; xzcdpc@126.com (X.S.); sunyonghong2528@163.com (Y.S.); 15895216226@163.com (D.L.); 4 School of Public Health, Nanjing Medical University, Nanjing 211166, China

**Keywords:** varicella vaccine, SV-1 cell line, adverse events following immunization, real-world study, safety monitoring, school-aged children

## Abstract

**Objectives**: Varicella is a highly contagious viral disease affecting children. The SV-1 cell line-based varicella attenuated live vaccine (SV-1VarV) is the first vaccine produced using the human diploid SV-1 cell substrate. This study evaluated the real-world safety of SV-1VarV among school-aged children in Jiangsu Province, China. **Methods**: A retrospective descriptive study was conducted using data from the Jiangsu Provincial Immunization Program Information System and the Chinese National Adverse Event Following Immunization Information System (CNAEFIS). Children aged 7–12 years who received SV-1VarV between July 2024 and March 2025 were included. The incidence, clinical characteristics, and demographic patterns of Adverse Events Following Immunization (AEFI) were analyzed. Reporting rates were calculated per 100,000 doses. Statistical analyses included chi-square tests, Cochran–Armitage trend tests, and Poisson regression analyses (α = 0.05). **Results**: A total of 366 AEFI cases were reported following 1,096,117 administered doses (33.4/100,000 doses), of which 364 were adverse reactions (33.2/100,000). General reactions accounted for 97.8% (mainly fever and local reactions), and abnormal reactions accounted for 2.2% (0.73/100,000). No serious adverse events or vaccine quality-related events occurred. Adverse reaction reporting rates declined with increasing age (*p* < 0.001) and were higher in males than females (36.7 vs. 29.2/100,000; *p* = 0.001). Poisson regression indicated that older age was independently associated with a lower risk of adverse reaction reporting, whereas sex and dose number were not significantly associated. **Conclusions**: SV-1VarV demonstrated a favorable safety profile during large-scale use in children aged 7–12 years. Most reactions were mild, self-limiting, and consistent with expected post-vaccination responses. These findings provide robust real-world evidence supporting the continued and expanded use of SV-1VarV in school-aged children to optimize varicella immunization strategies.

## 1. Introduction

Varicella, caused by the varicella-zoster virus (VZV), is an acute infectious disease transmitted through direct contact or respiratory droplets. It typically manifests with fever and a vesicular rash involving the skin and mucous membranes [1]. The disease is highly contagious in collective settings such as childcare centers and schools, posing a substantial burden on the health and education of children and adolescents [2] (pp. 154–196). Globally, according to the World Health Organization (WHO) and based on the 2019 Global Burden of Disease study, varicella was responsible for an estimated 83.96 million incident cases (95% uncertainty interval: 78.34–90.97 million) and 14,553 deaths (95% uncertainty interval: 12,909–17,103) annually, as summarized in the most recent WHO position paper on varicella vaccines [3] (pp. 569–570). In Europe, where routine childhood varicella vaccination has not been fully implemented, about 5.5 million cases occur annually, resulting in roughly 20,000 hospitalizations and 80 deaths [4]. Before the introduction of universal vaccination in the United States, around 4 million cases were reported annually, leading to over 10,000 hospitalizations and an average of 0.4 deaths per million population [5]. Following widespread vaccination, these figures declined markedly—to 1390 hospitalizations and 29 deaths per year between 2017 and 2019 [6]. Similarly, in Japan, annual varicella cases decreased from over 200,000 before 2013 to around 65,000 in 2016 after the adoption of a national immunization program [7].

In Jiangsu Province, China, 499 varicella-related public health emergencies were reported between 2014 and 2018, involving 13,177 cases and showing an increasing trend over time [8]. To reduce disease incidence and prevent outbreaks, the VarV was incorporated into the provincial immunization program in January 2023, implementing a two-dose schedule for children [9]. From July 2024 to March 2025, a province-wide free vaccination campaign targeting children aged 7–12 years was conducted to strengthen population immunity [10].

With the expanding use of VarV, its safety profile has become a public health priority. The SV-1VarV, developed using the SV-1 human diploid cell line, represents the world’s first varicella attenuated live vaccine based on this platform. According to WHO requirements, vaccine-producing cell substrates must undergo comprehensive characterization, including master and working cell bank establishment and validation for the absence of adventitious agents [11]. The SV-1 cell line, developed by Sinovac, has a well-documented origin, exhibits no tumorigenicity, and meets both national quality and WHO prequalification standards [12]. Clinical trials have confirmed the strong immunogenicity, efficacy, and safety of the SV-1 cell line–derived varicella vaccine [13].

Given that children aged 7–12 years are a key group for catch-up immunization, this study analyzed AEFI after large-scale administration of SV-1VarV in Jiangsu Province (July 2024–March 2025) to evaluate its real-world safety and provide evidence to optimize varicella vaccination strategies for school-aged populations.

## 2. Materials and Methods

### 2.1. Study Design

This study used a retrospective descriptive method to analyze the occurrence of AEFI among children aged 7–12 years in Jiangsu Province, China after large-scale catch-up vaccination with the SV-1VarV. The dose data of SV-1 VarV administered to children aged 7–12 years in Jiangsu Province from July 2024 to March 2025 were obtained from the Jiangsu Immunization Program Information System. Data on AEFI following SV-1 VarV vaccination were collected through a passive surveillance system, namely the CNAEFIS, which operates under the Chinese Disease Prevention and Control Information System (CDPCIS).

As this study involved secondary analysis of anonymized surveillance data, ethical approval was waived in accordance with national regulations, and informed consent from parents or guardians was not required.

### 2.2. Vaccines and Vaccination Procedures

SV-1VarV is produced by Sinovac (Dalian) Vaccine Technology Co., Ltd. (Dalian, China) and received marketing approval in China in 2022. Each dose contains no less than 3.3 log_10_ PFU of live attenuated varicella zoster virus (VZV, Oka strain). The Oka strain was originally obtained from the American Type Culture Collection (ATCC). As previously described, the vaccine is manufactured using Sinovac’s independently developed human diploid cell line, SV-1 [14]. SV-1VarV was administered via a 0.5 mL subcutaneous injection at the lower border of the deltoid muscle of the upper arm. Children who had not been vaccinated against varicella or had received only one dose were recommended to complete a two-dose varicella vaccination schedule, with an interval of at least three months between doses.

### 2.3. Study Setting and Population

This study focuses on children aged 7–12 years who meet the eligibility criteria for vaccination with the SV-1VarV. The inclusion criteria for the study population are as follows: Adolescents aged 7–12 years who completed 1 or 2 doses of SV-1VarV vaccination during the study period; Individuals whose vaccination records were successfully entered into the Jiangsu Provincial Comprehensive Vaccination Service Management Information System (JPCVs) by qualified vaccination clinics; Cases of AEFI that were reported and documented in the CNAEFIS [15]. The exclusion criteria are as follows: Cases with incomplete vaccination records or AEFI reports, or missing key information.

According to provincial demographic statistics, Jiangsu Province had approximately 2,196,603 children aged 7–12 years during province-wide catch-up vaccination campaign. During which 1,867,826 children completed a two-dose schedule of varicella vaccination, corresponding to a two-dose coverage rate of 85.1%. Among these fully vaccinated children, at least 700,708 received two doses of SV-1VarV, accounting for 37.5% of those who completed the two-dose schedule, while the remaining 62.5% received varicella vaccines produced by the other two manufacturers.

### 2.4. AEFI Reporting and Classification

According to the National Surveillance Program for Suspected Adverse Events Following Immunization [15], AEFI are reported by medical institutions and vaccination units, and verified by county-level Centers for Disease Control and Prevention (CDC). Except for cases clearly diagnosed as general reactions (e.g., simple fever, redness, or induration at the injection site), other AEFI are investigated by the corresponding level of CDC, with final diagnosis made by expert groups organized for AEFI investigation and diagnosis. Causality assessment of AEFI follows the World Health Organization (WHO)–recommended causality assessment algorithm (WHO–UMC system). Expert panels reviewed cases based on temporal association, biological plausibility, exclusion of alternative causes, and vaccine-related factors, and classified events into five types: adverse reactions (including general and abnormal reactions), vaccine quality–related events, immunization errors, coincidental events, and psychogenic reactions.

### 2.5. Statistical Analysis

All data were processed using Microsoft Excel 2019 and analyzed in R software (version 4.5.2). If multiple vaccines were suspected in a single case, the first listed vaccine was considered as VarV for analysis. Adverse reactions were classified according to the National AEFI Surveillance Guideline, and all abnormal reaction terms were coded using MedDRA version 26.1 Preferred Terms (PTs).

The reporting rate was calculated as: Reporting rate (/100,000 doses) = (Number of AEFI report cases/Number of SV-1VarV doses administered) × 100,000. Exact 95% confidence intervals (CIs) were estimated using the exact Poisson method.

Categorical variables were compared using the Pearson chi-square (χ^2^) test when expected cell counts ≥ 5 and the Fisher’s exact test when expected counts < 5. For ordered categorical variables, linear trends were evaluated using the Cochran–Armitage trend test (exact version).

Poisson regression with a log link and robust standard errors was used to estimate adjusted rate ratios (aRRs) and 95% CIs for demographic and vaccination characteristics. Covariates included sex, age (continuous), dose number (first vs. second), and city (entered as a fixed effect). The log of vaccine doses was used as an offset to account for exposure. Overdispersion was assessed by comparing residual deviance to degrees of freedom; robust standard errors were used to address mild overdispersion and potential heteroskedasticity. Poisson regression was conducted using adverse reaction counts (excluding coincidental and psychogenic events) as the outcome variable.

Model diagnostics indicated overdispersion in the Poisson model, with deviance divided by degrees of freedom (deviance/DF) is 1.35. Negative binomial (NB) regression provided a better fit as indicated by a lower Akaike Information Criterion (AIC) (ΔAIC = 12, Deviance/DF = 1.06), and yielded consistent effect estimates, supporting the robustness of the main findings.

To assess potential effect modification, an interaction term between age and sex (age × sex) was added to the Poisson regression model. The statistical significance of the interaction was evaluated using Wald tests with robust standard errors. The same interaction was further examined in the negative binomial model as a sensitivity analysis.

To control for Type I error due to multiple testing in descriptive analyses, false discovery rate (FDR) adjustment using the Benjamini–Hochberg procedure was applied to all chi-square and Cochran–Armitage trend tests. Regression analyses were prespecified and considered confirmatory; therefore, nominal two-sided *p* values are reported. Adjusted *p* values (q values) <0.05 were considered statistically significant.

All statistical tests were two-sided, and analyses were conducted in accordance with standard epidemiological and biostatistical practices for post-marketing vaccine safety surveillance.

## 3. Results

### 3.1. Basic Information

From July 2024 to March 2025, a total of 1,096,117 doses of SV-1VarV were administered to children aged 7–12 years in Jiangsu. In total, 366 AEFI cases were reported (33.39/100,000 doses), of which 364 were classified as adverse reactions (33.21/100,000 doses). General and abnormal reactions accounted for 356 (32.47/100,000) and 8 (0.73/100,000) cases, respectively; coincidental syndromes and psychogenic reactions were one case each (0.09/100,000 for both). No vaccine quality incidents or cluster events were reported.

### 3.2. Epidemiological Characteristics of Adverse Reactions

#### 3.2.1. Population Distribution

Adverse reaction reporting rates were higher in males (36.7/100,000 doses) than females (29.2/100,000 doses, *p* = 0.031). Adverse reaction incidence decreased steadily with age, peaking at 133.2/100,000 among 7-year-olds and lowest at 9.2/100,000 among 12-year-olds (*p* < 0.001) (Table 1).

#### 3.2.2. Dose Distribution

Among 390,584 first doses and 705,533 s doses, adverse reaction reporting rates were 34.8 (95% CI: 29.2–41.1) and 32.3 (95% CI: 28.3–36.8) per 100,000 doses for the first and second doses, respectively (*p* = 0.491) (Table 1).

#### 3.2.3. Regional Distribution

AEFI reporting rates differed significantly among 13 cities (χ^2^ = 148.40, df = 12, *p* < 0.001). Standardized residuals showed Zhenjiang (10.25) and Changzhou (4.51) reported rates markedly above expected, while Nanjing and Suzhou were below expected, likely reflecting heterogeneity in local surveillance sensitivity (Table 2).

#### 3.2.4. Poisson Regression Results

After adjustment for sex, dose number, and city fixed effects, age remained inversely associated with adverse reaction reporting (aRR = 0.64, 95% CI: 0.58–0.71, *p* < 0.001), indicating an average 36% decrease in reporting rate per additional year of age. Although the crude rate declined by about 90% from 7- to 12-year-olds, this adjusted estimate reflects the independent effect of age after controlling for potential confounders.

Although males showed a higher adjusted reporting rate than females (aRR = 1.27, 95% CI: 0.99–1.62), the 95% confidence interval included 1.0 and the association did not reach conventional statistical significance (*p* = 0.059), indicating limited evidence for a statistically or clinically meaningful sex difference. Dose number showed no significant association (aRR = 0.94, 95% CI: 0.72–1.23, *p* = 0.66). City fixed effects were jointly significant (*p* < 0.001), indicating residual between-city heterogeneity after covariate adjustment, consistent with the crude chi-square comparison across cities.

To evaluate whether the age-related decline in adverse reaction reporting differed by sex, an age × sex interaction term was included in the regression models. The interaction was not statistically significant in either the Poisson model (aRR = 1.09, 95% CI: 0.90–1.32; *p* = 0.37) or the NB model (aRR = 1.10, 95% CI: 0.91–1.33; *p* = 0.35), indicating that the inverse association between age and adverse reaction reporting was consistent across sexes.

#### 3.2.5. Model Diagnostics & Sensitivity Analysis

Model diagnostics revealed moderate overdispersion in the Poisson regression model (deviance/df = 1.35; Pearson dispersion = 2.34). NB regression was therefore conducted as a sensitivity analysis and demonstrated a clear improvement in model fit (ΔAIC = 12; deviance/df = 1.06). Although some residual dispersion remained, the negative binomial model substantially reduced extra-Poisson variability. Estimated rate ratios were highly consistent between the two models, indicating that the main findings were robust to alternative distributional assumptions (Supplementary Tables S1 and S2).

The absence of a statistically significant age × sex interaction in both models further supports the robustness and consistency of the main findings.

### 3.3. Clinical Diagnosis of Adverse Reactions

#### 3.3.1. General Reactions

Among general reactions, fever (45.9%) and redness/swelling (45.2%) were most common, followed by induration (23.9%). The rate of general reactions was 32.47/100,000 doses (95% CI: 29.4–35.7). The overall distribution of general reactions differed significantly (χ^2^ = 36.52, df = 2, *p* < 0.001). After FDR adjustment, the monotonic trends in fever severity and reaction size remained statistically significant.

Trend analysis showed that the proportion of fever increased significantly with higher temperature grades (*p* = 0.020), whereas no significant trend was observed across diameter groups for redness/swelling (*p* = 0.693) or induration (*p* = 0.880) (Table 3).

#### 3.3.2. Abnormal Reactions

Eight cases (0.73/100,000 doses, 95% CI, 0.32–1.44) were diagnosed as abnormal reactions, including rash allergic (n = 2), febrile convulsion (n = 2), Henoch–Schönlein purpura (n = 2), urticaria (n = 1), and one other event. No significant difference was found among subtypes of abnormal reactions (*p* = 0.990) (Table 3).

#### 3.3.3. Time of Onset and Outcome

Among 364 adverse reactions, the majority of events occurred within 3 days after vaccination. Specifically, among 356 general reactions, 196 cases (55.1%) occurred within 1 day, 149 (41.9%) within 3 days, and only 11 (3.1%) occurred ≥3 days post-vaccination. Of the eight abnormal reactions, four (50.0%) occurred within 1 day, one (12.5%) within 3 days, and three (37.5%) after ≥3 days. Febrile convulsion and convulsion occurred within 1 day, whereas Henoch–Schönlein purpura manifested after ≥3 days.

Fisher’s exact test comparing the time distribution between general and abnormal reactions indicated a statistically significant difference (*p* = 0.001), indicating an association between reaction type and onset timing.

Regarding clinical outcomes, most general reactions resolved (n = 319, 89.6%) or improved (n = 37, 10.4%). Among the eight abnormal reactions, six recovered completely (75.0%), one improved (12.5%), and one case remained under treatment at the time of reporting (Table 4).

No persistent sequelae or vaccine quality–related incidents were observed (Table 4).

## 4. Discussion

This study is the first to report the real-world safety of large-scale administration of SV-1 VarV among school-aged children (7–12 years). The findings indicate that the incidence of adverse reactions was low and within the expected range, demonstrating good overall safety.

In this study, the reported incidence rates of AEFI (33.39/100,000 doses) and adverse reactions (33.21/100,000 doses) following SV-1 VarV vaccination among children aged 7–12 years were lower than those reported for varicella vaccines in Jiangsu Province during 2021–2022 (46.83 and 44.91/100,000 doses, respectively) [16], likely because most vaccine recipients in that period were younger children aged 1–6 years. However, the rates observed here were slightly higher than the national averages for varicella vaccines in China during 2014–2020 (29.80 and 28.88/100,000 doses) [17] and in 2023 (32.33 and 31.87/100,000 doses) [18]. This may be related to the absence of varicella vaccine in the national EPI program, leading to lower coverage nationwide, while Jiangsu Province has implemented province-wide vaccination and this study involved large-scale supplementary immunization.

Among children aged 7–12 years, most AEFI following SV-1 VarV vaccination were general reactions (e.g., fever, redness, induration), accounting for 97.27% of all reported cases, representing typical post-vaccination reactogenic responses [19]. Fever was the most common reaction, reflecting normal immune activation in response to antigen recognition [20], with an incidence of 14.96/100,000 doses. High fever (axillary temperature ≥38.6 °C) accounted for 18.54% of general reactions and occurred mostly within one day after vaccination, which was slightly lower than the latest post-marketing surveillance data reported by Merck (approximately 20/100,000 doses) [21]. Therefore, vaccination points (POVs) and healthcare providers should strengthen scientific communication and provide clear guidance on fever management to ease parental concern and reduce the risk of febrile convulsions.

The abnormal reaction rate following SV-1 VarV vaccination was 0.73/100,000 doses, mainly involving allergic rash, febrile convulsion, allergic purpura, and urticaria. This rate was lower than that reported in Jiangsu Province during 2021–2022 (1.60/100,000 doses) [16] and the national average for varicella vaccines from 2014–2020 (3.12/100,000 doses) [17]. It was comparable to the national level in 2023 (0.73/100,000 doses) [18] and well below the WHO global reference threshold for vaccine safety monitoring (<1/10,000) [22].

All abnormal reactions were mild to moderate and were generally resolved by the time of reporting. Most occurred within one day after vaccination, consistent with the findings of Shi Lubin et al. [23], mainly reflecting short-term reactogenicity. Although abnormal reactions were more frequently observed at later time points after vaccination, this finding reflects a statistical association in temporal patterns and should not be interpreted as evidence of causality. During the monitoring period, no severe abnormal reactions previously reported nationwide for varicella vaccines between 2014 and 2020 were observed, such as anaphylactic shock, encephalitis or meningitis, injection site abscess, acute disseminated encephalomyelitis, lymphadenitis, injection site necrosis, Guillain–Barré syndrome, erythema multiforme, encephalopathy, or thrombocytopenic purpura [17].

A Poisson regression model was used to quantitatively assess factors associated with adverse reaction reporting rates. The analysis showed that with each one-year increase in age, the reporting rate decreased by approximately 36%, indicating a significant decline in risk with age. This pattern aligns with previous findings from varicella vaccine safety monitoring in China, showing that adverse reactions, particularly local inflammation and fever, decrease with increasing age [17,24,25]. The observed age-related decline in adverse reaction reporting is biologically plausible and may reflect maturational changes in the immune system. Younger children tend to mount stronger innate and adaptive immune responses to live attenuated vaccines, which are often associated with higher reactogenicity, such as fever and local inflammatory reactions. With increasing age, immune responses become more regulated, potentially resulting in reduced reactogenic manifestations. Mechanistic studies have shown that age-related changes in dendritic cell function, interferon signaling, and inflammatory regulation can influence vaccine-induced reactogenicity without necessarily compromising immunogenicity [19,24,25].

The observed sex difference in adverse reaction reporting was modest and did not reach statistical significance after adjustment, with the 95% CI overlapping unity. This suggests limited evidence for a clinically meaningful sex-related difference. The observed between-city heterogeneity is more likely to reflects differences in local surveillance sensitivity, reporting practices, and local implementation of AEFI monitoring, rather than true differences in vaccine safety. No significant difference was found between the first and second doses. Overall, the model results confirmed that the risk of adverse reactions was low and mainly influenced by age and sex, indicating stable safety of SV-1 VarV across subpopulations without dose-related risk or regional clustering. Moreover, no evidence of effect modification by sex was observed, suggesting that the age-related decline in vaccine reactogenicity operates similarly in boys and girls.

Although Poisson regression is commonly used for modeling AEFI reporting rates, evidence of overdispersion was observed in the present analysis. Negative binomial regression substantially improved model fit and yielded effect estimates consistent with the primary Poisson analysis. This supports the robustness of the observed associations and suggests that the findings are not driven by model misspecification

This study has several limitations. First, it was based on passive surveillance data from JPCVs and CNAEFIS, which are subject to inherent limitations such as underreporting, reporting delays, and variability in reporting completeness and diagnostic accuracy across regions and healthcare facilities. AEFI rates are likely to underestimate the true incidence of adverse events following immunization, and causal inference is inherently limited. Therefore, the findings should be interpreted as reflecting reported safety signals under routine surveillance conditions rather than the absolute incidence of all adverse events.

However, the large sample size and province-wide coverage provide strong real-world evidence on the post-marketing safety of SV-1 VarV. Under this framework, the AEFI reporting rate was low, and no quality-related events were identified. Future studies incorporating active surveillance or multi-province data linkage are needed to validate rare AEFI findings and improve detection power. Although direct comparison with varicella vaccines produced using other cell lines was not performed, it is notable that before 2022—the year the Sinovac varicella vaccine was introduced—most varicella vaccines used in Jiangsu were derived from other diploid cell lines (2BS and MRC-5). Thus, the results provide supportive evidence for the safety of SV-1 VarV relative to these vaccines.

Although capture–recapture methods have been proposed to estimate the true incidence of AEFI by adjusting for underreporting, the current study was limited by the availability of only a single passive AEFI reporting system, precluding the application of such methods. Future studies integrating multiple independent data sources or active surveillance systems may allow for more accurate incidence estimation.

The findings have important implications for varicella vaccination policy. Given the good safety profile of SV-1 VarV in large-scale immunization among school-aged children, expanding its use could enhance herd immunity and reduce varicella burden. Integrating SV-1 VarV into routine or catch-up immunization programs, while strengthening post-marketing surveillance and inter-provincial data sharing, would help maintain high vaccine coverage, ensure early detection of rare events, and support evidence-based decisions for optimizing the national immunization program.

## 5. Conclusions

This study provides the first large-scale real-world evidence on the safety of SV-1 VarV in children aged 7–12 years. The overall incidence of AEFI was low, with most reactions mild, short-term, and consistent with expected vaccine responses. No serious safety concerns were identified. Both descriptive and regression analyses showed that AEFI risk decreased with age and remained stable across subpopulations, confirming the favorable safety profile of SV-1 VarV. These results support its continued and expanded use among school-aged children and its inclusion in broader immunization programs.

## Figures and Tables

**Table 1:** Age, Sex, and Dose Distribution of Adverse Reactions to SV-1VarV.

Category	General Reactions		Abnormal Reactions		Total Adverse Reactions			
	Cases	Rates	Cases	Rates	Cases	Rates	95% CI	*p* Value
Sex								
Male	209	35.85	5	0.86	214	36.7	32.0, 42.0	0.001
Female	147	28.65	3	0.58	150	29.2	24.7, 34.3	
Age (years)								0.001
7	23	127.64	1	5.55	24	133.2	85.3, 198.2	
8	68	83.59	3	3.69	71	87.3	68.2, 110.1	
9	71	35.99	1	0.51	72	36.5	28.6, 46.0	
10	91	33.41	2	0.73	93	34.1	27.6, 41.8	
11	77	29.04	1	0.38	78	29.4	23.3, 36.7	
12	26	9.93	0	0	26	9.9	6.5, 14.5	
Dose								0.491
1st Dose	133	34.05	3	0.77	136	34.8	29.2, 41.2	
2nd Dose	223	31.61	5	0.71	228	32.3	28.3, 36.8	

Rates are per 100,000 doses. *p* values for overall comparisons (parent rows) were adjusted for multiple comparisons using the BH FDR procedure.

**Table 2:** Adverse Reactions to SV-1VarV by City in Jiangsu Province.

City	Cases	Proportion (%)	Doses	Rates	95% CI	Expected Cases ^a^	Std. Residual ^b^
Nanjing	6	1.65	43,483	13.8	5.06, 30.03	14.44	−2.22
Wuxi	6	1.65	36,384	16.49	6.05, 35.89	12.08	−1.75
Xuzhou	91	25.00	329,058	27.65	22.27, 33.95	109.27	−1.75
Changzhou	44	12.09	67,950	64.75	47.05, 86.93	22.56	4.51
Suzhou	18	4.95	90,351	19.92	11.81, 31.49	30.00	−2.19
Nantong	18	4.95	81,047	22.21	13.16, 35.10	26.91	−1.72
Lianyungang	18	4.95	70,773	25.43	15.07, 40.20	23.50	−1.14
Huai’an	22	6.04	74,402	29.57	18.53, 44.77	24.71	−0.54
Yancheng	33	9.07	94,021	35.10	24.16, 49.30	31.22	0.32
Yangzhou	10	2.75	41,348	24.18	11.60, 44.48	13.73	−1.01
Zhenjiang	50	13.74	39,209	127.52	94.65, 168.12	13.02	10.25
Taizhou	25	6.87	58,919	42.43	27.46, 62.64	19.57	1.23
Suqian	23	6.32	69,172	33.25	21.08, 49.89	22.97	0.01

^a^ Expected Cases = (Total Cases × City doses/Total doses); ^b^ Std. Residual = (Observed − Expected)/√Expected; Rates are per 100,000 doses.

**Table 3:** Clinical Diagnosis Distribution of Adverse Reactions to SV-1VarV in Children Aged 7–12 Years in Jiangsu Province.

Clinical Diagnosis	Cases	Rates	95% CI	*p* Value
General Reactions				
Fever	162	14.96	12.74, 17.41	<0.001
37.1–37.5 °C	22	2.01	1.26, 3.04	
37.6–38.5 °C	75	6.93	5.46, 8.68	
≥38.6 °C	65	6.02	4.66, 7.66	
Redness and swelling	161	14.69	12.52, 17.13	<0.001
≤2.5 cm	49	4.47	3.31, 5.91	
2.6–5.0 cm	83	7.57	6.03, 9.39	
>5.0 cm	29	2.65	1.77, 3.80	
Induration	85	7.75	6.17, 9.57	<0.001
≤2.5 cm	32	2.92	2.00, 4.12	
2.6–5.0 cm	41	3.74	2.68, 5.07	
>5.0 cm	12	1.09	0.57, 1.91	
Other symptoms	42	3.83	2.76, 5.18	-
Abnormal Reactions				
Allergic Rash	2	0.18	0.02, 0.65	1.000
Febrile Convulsion	1	0.09	0.00, 0.51	
Convulsion	1	0.09	0.00, 0.51	
Urticaria	1	0.09	0.00, 0.51	
Henoch–Schönlein purpura	2	0.18	0.02, 0.65	
Central nervous system demyelinating disease (MOGAD)	1	0.09	0.00, 0.51	

Rates are per 100,000 doses. *p* values for overall comparisons (parent rows) were adjusted for multiple comparisons using the BH FDR procedure.

**Table 4:** Time Distribution of Adverse Reactions.

Adverse Reaction Category	Number of Reports	<1 d		<3 d		≥3 d	
		Cases	Composition Ratio (%)	Cases	Composition Ratio (%)	Cases	Composition Ratio (%)
General Reactions	356	196	55.06	149	41.85	11	3.09
Abnormal Reactions	8	4	50.00	1	12.50	3	37.50
Allergic Rash	2	1	50.00	1	50.00	—	—
Febrile convulsion	1	1	100.00	—	—	—	—
convulsion	1	1	100.00	—	—	—	—
Urticaria	1	1	100.00	—	—	—	—
Henoch–Schönlein purpura	2	—	—	—	—	2	100.00
Central nervous system demyelinating disease (MOGAD)	1	1	100.00	—	—	1	100.00

## Data Availability

The data presented in this study are available on request from the corresponding authors. The data are not publicly available due to privacy restrictions.

## Supplementary Materials

The following supporting information can be downloaded at https://www.mdpi.com/article/10.3390/vaccines14010019/s1, Table S1: Comparison of aRRs estimated from Poisson and NB regression models; Table S2: Model fit diagnostics for Poisson and negative binomial regression models.

## Abbreviations

The following abbreviations are used in this manuscript:

SV-1VarV	SV-1 Cell Line-Based Varicella Attenuated Live Vaccine
AEFI	Adverse Events Following Immunization
VZV	Varicella-Zoster Virus
WHO	World Health Organization
NHIS	National Health Interview Survey
CDC	Centers for Disease Control and Prevention
JPCVs	Jiangsu Provincial Comprehensive Vaccination Service Management Information System
CNAEFIS	Adverse Event Following Immunization Information System
CDPCIS	Chinese Disease Prevention and Control Information System
ATCC	American Type Culture Collection
PTs	Preferred Terms
FDR	False Discovery Rate
POV	Point of Vaccination
