# Supplementary material for: Safety Evaluation of Large-Scale Administration of a Novel Human Diploid (SV-1) Cell Line-Derived Varicella Attenuated Live Vaccine in Children 7–12 Years Old
Source: Vaccines (Basel). 2025 Dec 23;14(1):19. doi: 10.3390/vaccines14010019 (PMC12846628; doi:10.3390/vaccines14010019)
Supplement: Supplementary file 1 [file vaccines-14-00019-s001.zip › vaccines-4044301-supplementary.pdf]

Table S1. Comparison of aRRs estimated from Poisson and NB regression models.

	Poisson regression					NB regression				
**Variable**	**aRR**	**95% CI**	**Robust SE**	**z**	**P**	**aRR**	**95% CI**	**SE**	**z**	**P**
Male (vs Female)	1.27	0.99–1.62	0.13	1.89	0.059	1.31	1–1.7	0.13	1.99	0.046
Age (per year)	0.64	0.58–0.71	0.05	-8.34	<0.001	0.61	0.55–0.68	0.05	-9.31	<0.001
Age × Male (interaction)	1.09	0.9–1.32	0.10	0.90	0.370	1.10	0.91–1.33	0.10	0.94	0.347
2nd dose (vs 1st)	0.94	0.72–1.23	0.14	-0.44	0.662	0.87	0.66–1.16	0.15	-0.93	0.351
city Changzhou	2.07	1.29–3.32	0.24	3.01	0.003	2.20	1.37–3.55	0.24	3.25	0.001
city Huai'an	0.89	0.49–1.61	0.31	-0.39	0.693	0.92	0.51–1.68	0.30	-0.26	0.795
city Lianyungang	0.77	0.41–1.43	0.32	-0.83	0.405	0.76	0.4–1.43	0.32	-0.85	0.394
city Nanjing	0.34	0.17–0.68	0.35	-3.06	0.002	0.34	0.17–0.69	0.37	-2.96	0.003
city Nantong	0.70	0.42–1.17	0.26	-1.35	0.176	0.71	0.42–1.2	0.27	-1.29	0.197
city Suzhou	0.73	0.4–1.35	0.31	-1.00	0.316	0.72	0.39–1.36	0.32	-1.01	0.312
city Taizhou	1.38	0.79–2.43	0.29	1.13	0.256	1.36	0.77–2.39	0.29	1.07	0.283
city Wuxi	0.68	0.24–1.92	0.53	-0.73	0.463	0.68	0.24–1.93	0.53	-0.72	0.473
city Xuzhou	0.81	0.5–1.32	0.25	-0.85	0.393	0.93	0.56–1.53	0.26	-0.30	0.768
city Yancheng	1.55	0.9–2.68	0.28	1.58	0.113	1.82	0.99–3.34	0.31	1.93	0.053
city Yangzhou	0.77	0.3–1.98	0.48	-0.55	0.581	0.79	0.31–2.01	0.48	-0.50	0.615
city Zhenjiang	3.70	2.18–6.26	0.27	4.87	<0.001	4.21	2.28–7.79	0.31	4.58	<0.001

Notes: adjusted rate ratios (aRRs) and 95% confidence intervals (CIs) from Poisson regression with robust standard errors and negative binomial regression are presented. Models were adjusted for sex, age, dose number, city, and an interaction term between age and sex, with vaccine doses included as an offset. Consistent estimates across models suggest robustness of the results to potential overdispersion.

Table S2. Model fit diagnostics for Poisson and negative binomial regression models.

Model	AIC	Residual Deviance	Residual DF	Deviance/DF	Pearson dispersion (φ)
Poisson	849.0147	399.5644	296	1.3499	2.3415
NB	836.8978	312.3978	296	1.0554	1.7712

Notes: AIC = Akaike Information Criterion. Deviance/DF and Pearson's dispersion parameter (φ) values greater than 1 indicate overdispersion. Lower AIC values indicate better model fit.
